# Updated MS²PIP web server supports cutting-edge proteomics applications

**DOI:** 10.1093/nar/gkad335

**Published:** 2023-05-04

**Authors:** Arthur Declercq, Robbin Bouwmeester, Cristina Chiva, Eduard Sabidó, Aurélie Hirschler, Christine Carapito, Lennart Martens, Sven Degroeve, Ralf Gabriels

**Affiliations:** VIB-UGent Center for Medical Biotechnology, VIB, Belgium; Department of Biomolecular Medicine, Ghent University, Belgium; VIB-UGent Center for Medical Biotechnology, VIB, Belgium; Department of Biomolecular Medicine, Ghent University, Belgium; Proteomics Unit, Universitat Pompeu Fabra, 08003, Barcelona, Spain; Proteomics Unit, Centre for Genomic Regulation, Barcelona Institute of Science and Technology (BIST), 08003, Barcelona, Spain; Proteomics Unit, Universitat Pompeu Fabra, 08003, Barcelona, Spain; Proteomics Unit, Centre for Genomic Regulation, Barcelona Institute of Science and Technology (BIST), 08003, Barcelona, Spain; Laboratoire de Spectrométrie de Masse BioOrganique (LSMBO), Université de Strasbourg, CNRS, France; Laboratoire de Spectrométrie de Masse BioOrganique (LSMBO), Université de Strasbourg, CNRS, France; VIB-UGent Center for Medical Biotechnology, VIB, Belgium; Department of Biomolecular Medicine, Ghent University, Belgium; VIB-UGent Center for Medical Biotechnology, VIB, Belgium; Department of Biomolecular Medicine, Ghent University, Belgium; VIB-UGent Center for Medical Biotechnology, VIB, Belgium; Department of Biomolecular Medicine, Ghent University, Belgium

## Abstract

Interest in the use of machine learning for peptide fragmentation spectrum prediction has been strongly on the rise over the past years, especially for applications in challenging proteomics identification workflows such as immunopeptidomics and the full-proteome identification of data independent acquisition spectra. Since its inception, the MS²PIP peptide spectrum predictor has been widely used for various downstream applications, mostly thanks to its accuracy, ease-of-use, and broad applicability. We here present a thoroughly updated version of the MS²PIP web server, which includes new and more performant prediction models for both tryptic- and non-tryptic peptides, for immunopeptides, and for CID-fragmented TMT-labeled peptides. Additionally, we have also added new functionality to greatly facilitate the generation of proteome-wide predicted spectral libraries, requiring only a FASTA protein file as input. These libraries also include retention time predictions from DeepLC. Moreover, we now provide pre-built and ready-to-download spectral libraries for various model organisms in multiple DIA-compatible spectral library formats. Besides upgrading the back-end models, the user experience on the MS²PIP web server is thus also greatly enhanced, extending its applicability to new domains, including immunopeptidomics and MS3-based TMT quantification experiments. MS²PIP is freely available at https://iomics.ugent.be/ms2pip/.

## Graphical Abstract

**Graphical Abstract:**
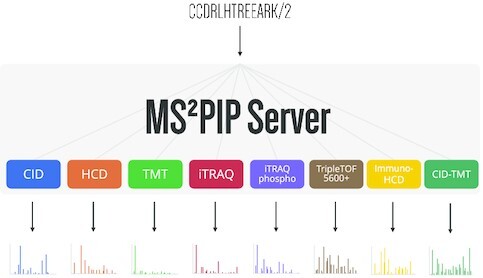
Updated MS²PIP web server supports cutting-edge proteomics applications.

## INTRODUCTION

Over the past decade, the ever-broadening scope of diverse proteomics workflows has engendered greatly increased interest in the field. However, these new applications each come with their specific challenges. For example, immunopeptidomics must address the non-tryptic nature of immunopeptides, whereas isobaric labelling for quantification can result in reduced identification efficiency (1,2). These specialized approaches, which build on sample preparation and proteomics acquisition innovations, therefore also require novel developments in data analysis to maximally exploit the value of the corresponding data.

One data analysis innovation that has impacted nearly all of the new proteomics workflows is the machine learning-based prediction of accurate peptide fragmentation spectra, as pioneered by MS²PIP and others (3,4). Indeed, we have previously showcased the wide applicability of MS²PIP predictions (5–7) (https://iomics.ugent.be/ms2pip), and how these can be leveraged to boost the yields from various proteomics identification strategies (8). Interesting use cases of these predictions include the rescoring of peptide-spectrum matches (PSMs) (8,9), the creation of proteome-wide spectral libraries for data-independent acquisition (DIA) (10,11) and streamlining the design of targeted proteomics experiments (12,13). While MS²PIP already supported a wide variety of fragmentation methods, instruments, and labelling techniques, the development of various novel impactful proteomics workflows resulted in a clear demand for additional, specialized MS²PIP models.

We have therefore further expanded MS²PIP with the requisite new prediction models, which now include support for tryptic- and non-tryptic peptides, for immunopeptides, and for collision-induced dissociation (CID) spectra of peptides treated with tandem-mass-tag (TMT) quantification labels. These new models allow MS²PIP to be applied in alternative digestion experiments, in immunopeptidomics experiments, and in MS3-TMT-based quantification studies. We have updated the MS²PIP web server to include these new prediction models, alongside several new features, such as the integration of our state-of-the-art retention time predictor DeepLC (14), the option to generate proteome-wide spectral libraries starting from only a FASTA file, and the availability of prebuilt, ready-to-download spectral libraries for ten common model organisms in multiple DIA-compatible file formats. These updates will further streamline downstream use of MS²PIP, allowing even wider adoption and utility.

## NEW IN THE 2023 VERSION OF THE MS²PIP WEB SERVER

### Updated MS²PIP core library with increased availability

Since the previous MS²PIP web server publication, we have drastically improved the availability and usability of MS²PIP’s core library. It is now available as a standalone Python package that can be easily installed on all major OS platforms with PyPI, with Bioconda, or as a BioContainer. In addition to the command line interface (CLI), a new Python interface now allows MS²PIP to be easily integrated into other tools and workflows. To compute correlations between observed and predicted spectra, MS²PIP now supports both MGF and mzML spectrum file formats. MS²PIP now also seamlessly integrates the state-of-the-art retention time predictor DeepLC. Furthermore, we have implemented two new operating modes for MS²PIP: (i) the *fasta2speclib* command allows users to generate proteome-wide predicted spectral libraries, starting from only a FASTA proteome file and (ii) the *single-prediction* command allows users to quickly predict a single spectrum directly from the CLI. The MS²PIP core package is open-source under the permissive Apache2 license, and is freely available at https://github.com/compomics/ms2pip/.

### Extended and improved MS²PIP web server

For an optimal, user-friendly experience, MS²PIP is made available as an online web server. Since its previous publication, we have significantly extended the MS²PIP web server functionality. First, the web server contains all new features of the MS²PIP core library, most notably including the new prediction models (see below). Second, without any additional configuration, users can opt to include accurate retention time predictions in the predicted libraries from our retention time predictor DeepLC. Third, the web server now also accepts—next to the existing peptide list input—a protein FASTA file with ‘search space’ settings that define which peptides will be included in the library. Configurable settings include the cleavage rules for *in silico* digestion, the number of allowed missed cleavages, the precursor *m/z* range, and common residue modifications. Fourth, we now provide ready-to-download spectral libraries for ten common model organism UniProt reference proteomes, including *Homo sapiens*, *Escherichia coli* and *Arabidopsis thaliana*. Each library is available in the MSP and SSL/MS2 file formats, ensuring compatibility with major DIA search engines, such as DIA-NN (15) and Skyline (16).

### New prediction models for (non-)tryptic peptides, immunopeptides and MS3 quantification experiments

We have updated MS²PIP with three new prediction models. The 2019 model for HCD fragmentation was originally only trained on tryptic peptides. Non-tryptic peptides, however, lack the basic lysine or arginine on their C-terminus, which heavily influences fragmentation patterns (17). As a result, the existing MS²PIP models performed sub optimally for non-tryptic peptides. To allow MS²PIP to be applied to proteomics workflows that yield non-tryptic peptides, such as alternative-digestion and biopeptidomics experiments, we have trained a new and improved HCD model capable of both tryptic and non-tryptic peptide predictions. This model was validated on external evaluation data sets containing peptides from both trypsin- and chymotrypsin-digestion. Importantly, this new, much more generic model outperforms the previous model on both tryptic and non-tryptic peptides. Additionally, we have trained a specialized model for immunopeptides to be used in immunopeptidomics experiments. This model was validated on both HLA class I and HLA class II peptides.

In quantitative mass spectrometry, MS3 acquisition of TMT-labeled spectra has been gaining popularity over traditional MS2 acquisition (18). However, the combination of CID fragmentation, ion trap acquisition of MS2 spectra, and of TMT-labelling substantially alters fragmentation patterns, which is detrimental for the performance of both the existing CID and HCD-TMT MS²PIP models. Therefore, we have trained and validated a new CID-TMT model to allow for applications of MS²PIP in MS3-TMT-based quantification studies.

Train, test, and evaluation data was downloaded from PRIDE (19,20) and converted to MS²PIP input files (Supplementary Table S1)—except for the CID-TMT training data, which was generated in-house and is available from PRIDE with identifier PXD041002 (see supplementary methods). While not explicitly considered for intensity prediction, the train and test data also included common modifications such as oxidation of M, carbamidomethylation of C and acetylation of the amino termini. To guarantee fully external unseen evaluation data sets, overlapping peptidoforms between train and test sets were removed from the test set. Similar to the 2019 MS²PIP models (7) all new models were trained with a gradient boosting machine learning algorithm (see Supplementary Table S2) as implemented in the XGBoost Python package (see supplementary methods).

### Performance of the new MS²PIP models

**Figure 1.**
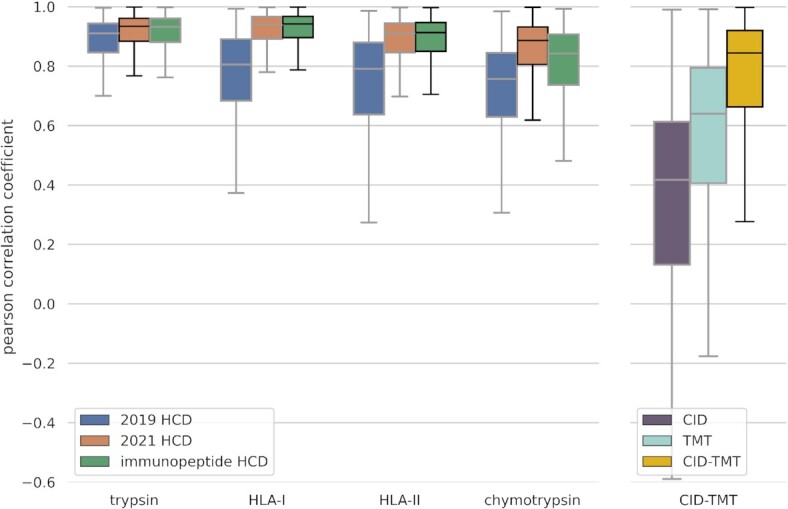
Distribution of Pearson correlation coefficients per spectrum (y-axis) for each newly trained model and the relevant existing models, evaluated on various external unseen data sets (x-axis). Each color represents a model, with the target model for each evaluation data set shown with black borders and the other models shown with grey borders.

To evaluate the newly added MS²PIP models, we selected several unseen, external evaluation data sets to compare the predictions with observed spectra and calculate Pearson correlation coefficients (PCC) per spectrum. The selected orbitrap HCD data sets consist of trypsin-digested, chymotrypsin-digested, HLA class I and HLA class II peptides, respectively. We compared the performance of the new MS²PIP HCD and immunopeptide models with the 2019 MS²PIP model on each of these evaluation data sets. Both new models showed substantial increases in performance on their respective target data sets, with a median PCC of 0.93 and 0.88 for the 2021 HCD model on trypsin and chymotrypsin and a median PCC of 0.94 and 0.91 for the immunopeptide HCD model on HLA-I and HLA-II data (Figure 1). Notably, even when evaluated on a trypsin-digested peptide data set, the new, more generic HCD model still shows an increase in performance, suggesting that combining tryptic and non-tryptic data for training leads to an overall better generalized model. Furthermore, the lower performance of the specialized immunopeptide model on chymotrypsin-digested peptide data indicates that these two types of non-tryptic peptides are likely very different. Indeed, separating predictive performance by peptide length shows a significant drop in accuracy for peptides longer than 17 amino acids for the immunopeptide model, while the new general HCD model shows a consistently high performance across peptide lengths (Supplementary Figure S1). When examining the prediction accuracies for HLA type I and type II in a similar manner, we observe an improved performance across all peptide lengths and for both HLA types, compared to the 2019 HCD model (Supplementary Figure S2).

Previously we have shown that acquisition modes and isobaric labelling techniques can heavily alter peak intensity patterns (7). This is especially the case for ion trap-based CID acquisition of TMT-labelled spectra. Evaluation on a CID-TMT data set shows that neither the existing CID nor the existing HCD-TMT MS²PIP models generalized well for this type of peptide spectra. Interestingly, the HCD-TMT model still outperforms the CID model, suggesting that the labelling method has a larger influence on peak intensity patterns than the fragmentation method (Supplementary Figure S3). This can be confirmed by correlating observed spectra directly for each type. Indeed, observed HCD-TMT spectra correlate slightly better with CID-TMT spectra than with unlabeled CID peptide spectra. Nevertheless, as both correlations are low, there was a need for a specialized CID-TMT prediction model. The newly trained CID-TMT model vastly outperforms current models with a median PCC of 0.84 (Figure 1).

## CONCLUSION AND FUTURE PERSPECTIVES

The use of machine learning-based predictive models for analyte behavior has become an indispensable part of proteomics, as is reflected by the number and popularity of machine learning tools – including MS²PIP – that have been published in the past years (3,4). Among these tools, the prediction of fragment intensities and peptide retention times have proven highly valuable useful to improve the confidence in peptide identification (9). While recently many deep learning-based spectrum predictors have been developed, the use of the gradient tree boosting (XGBoost) machine learning algorithm allows us to easily build accurate prediction models for specialized use cases where less training data might be available. Additionally, MS²PIP does not require graphical processing units and can be run on virtually any computer system. Nevertheless, with the updated MS²PIP web server we aim to make both MS²PIP and DeepLC even more easily accessible to the entire proteomics community. The updated MS²PIP web server is the first to allow users to generate proteome-wide spectral libraries on-the fly directly from a FASTA file and additionally provides pre-built spectral libraries for ten model organisms. Furthermore, thanks to the addition of three new, highly performant peptide spectrum prediction models, MS²PIP continues to support and push forward innovations in proteomics and its various established and emerging subfields.

## DATA AVAILABILITY

The MS²PIP web server is freely available at https://iomics.ugent.be/ms2pip/. The core library is open source, licensed under the permissive Apache-2.0 license, available as a package on PyPI, Bioconda, and BioContainers, and hosted on https://github.com/compomics/ms2pip/ and published on https://doi.org/10.5281/zenodo.7669701. All scripts for model training, evaluation, and figure generation are available on https://github.com/compomics/ms2pip/tree/v3.11.0/manuscripts/2023/. All training and evaluation data is available on Zenodo at https://doi.org/10.5281/zenodo.7669701. The newly generated CID-TMT data is available from PRIDE with identifier PXD041002 (https://www.ebi.ac.uk/pride/archive/projects/PXD041002).

## Supplementary Material

gkad335_Supplemental_File
