# Supplementary material for: Updated MS²PIP web server supports cutting-edge proteomics applications
Source: Nucleic Acids Res. 2023 May 4;51(W1):W338–42. doi: 10.1093/nar/gkad335 (PMC10320101; doi:10.1093/nar/gkad335)
Supplement: gkad335_Supplemental_File [file gkad335_supplemental_file.docx]

Supplementary to

Extended MS²PIP web server enables new proteomics applications

Arthur Declercq^1,2^, Robbin Bouwmeester^1,2^, Cristina Chiva^3,4^, Eduard Sabidó^3,4^, Aurélie Hirschler^5^,

Christine Carapito^5^, Lennart Martens^1,2^, Sven Degroeve^1,2 §^, and Ralf Gabriels^1,2^

^1^ VIB-UGent Center for Medical Biotechnology, VIB, Belgium
^2^ Department of Biomolecular Medicine, Ghent University, Belgium

^3^ Proteomics Unit, Universitat Pompeu Fabra, 08003 Barcelona, Spain

^4^ Proteomics Unit, Centre for Genomic Regulation, Barcelona Institute of Science and Technology (BIST), 08003 Barcelona, Spain

^5^ Laboratoire de Spectrométrie de Masse BioOrganique (LSMBO), Université de Strasbourg, CNRS, France

^§^ To whom correspondence should be addressed:
Tel: +32 9 224 98 54
Email: [sven.degroeve@vib-ugent.be](mailto:sven.degroeve@vib-ugent.be)
Address: Technologiepark 75, 9052 Ghent, Belgium

ORCID IDs:
Arthur Declercq: [https://orcid.org/0000-0002-9376-1399](https://orcid.org/0000-0002-9376-1399/)
Robbin Bouwmeester: [https://orcid.org/0000-0001-6807-7029](https://orcid.org/0000-0001-6807-7029/)

Cristina Chiva: <https://orcid.org/0000-0001-8150-6203>

Eduard Sabidó: <https://orcid.org/0000-0001-6506-7714>
Aurélie Hirschler: [https://orcid.org/0000-0001-5066-6263](https://orcid.org/0000-0001-5066-6263/)
Christine Carapito: <https://orcid.org/0000-0002-0079-319X>
Lennart Martens: <https://orcid.org/0000-0003-4277-658X>
Sven Degroeve: <https://orcid.org/0000-0001-8349-3370>
Ralf Gabriels: <https://orcid.org/0000-0002-1679-1711>

# Supplementary information

## Supplementary figures


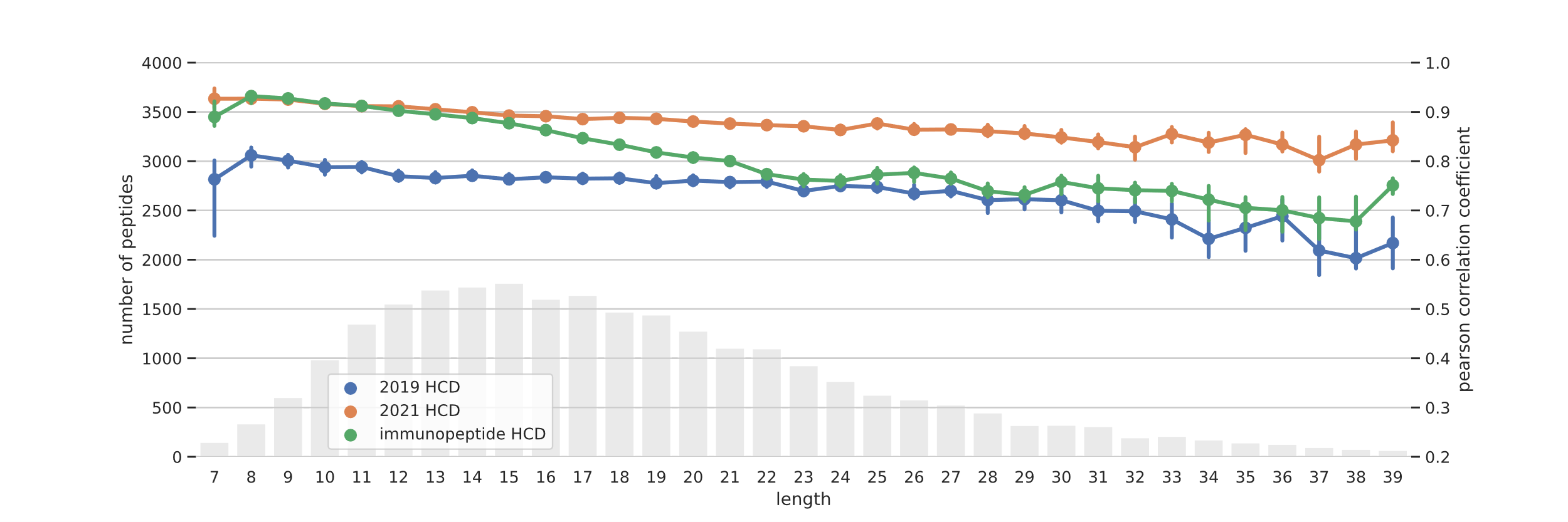


Figure S1. Distribution of Pearson correlation coefficients on the chymotrypsin evaluation data set separated by peptide length, where the grey bars indicate the number of peptides for each length.


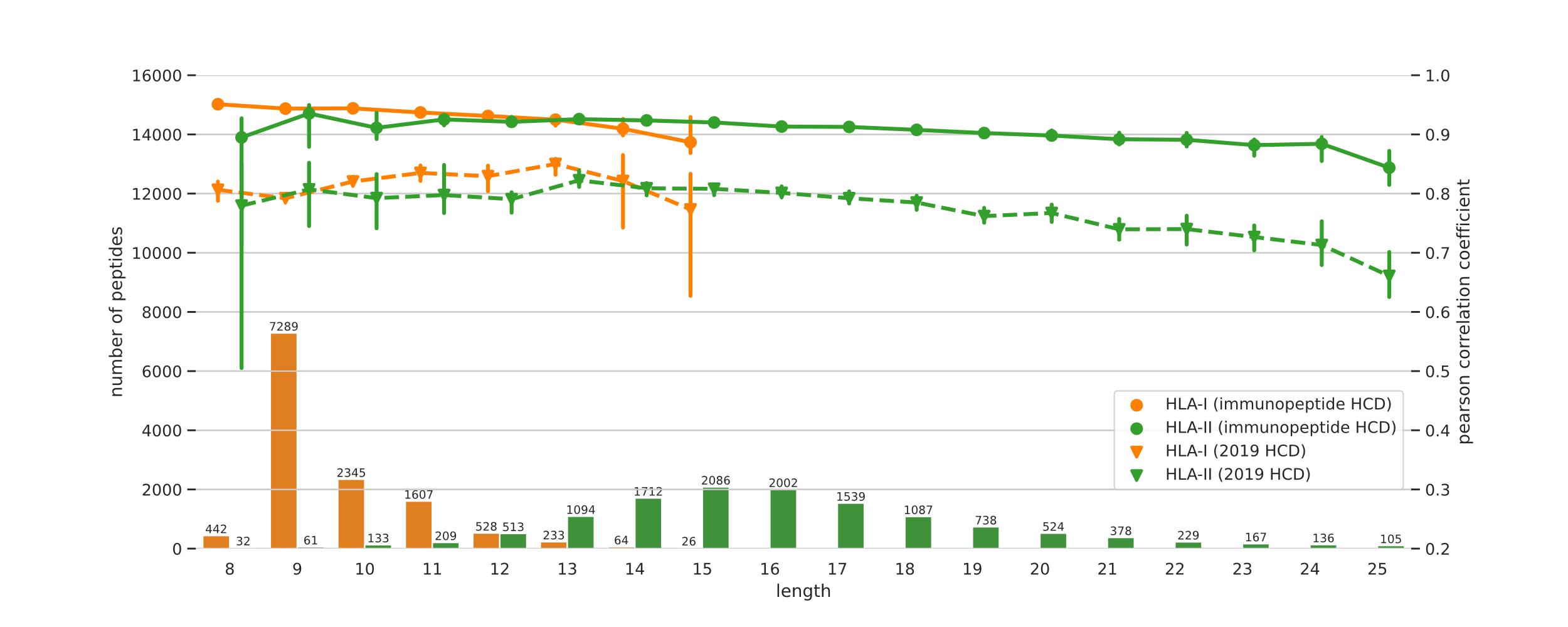


Figure S2. Distribution of Pearson correlation coefficients on the HLA-I (orange) and HLA-II (green) evaluation data set separated by peptide length, where the bars indicate the number of HLA-I (orange) and HLA-II (green) peptides for each length. The full line with the round markers denotes the Pearson correlation coefficients for the predictions of the new immunopeptide HCD model, and the dashed line with triangle markers denotes the Pearson correlation coefficients for the predictions of the 2019 HCD model.


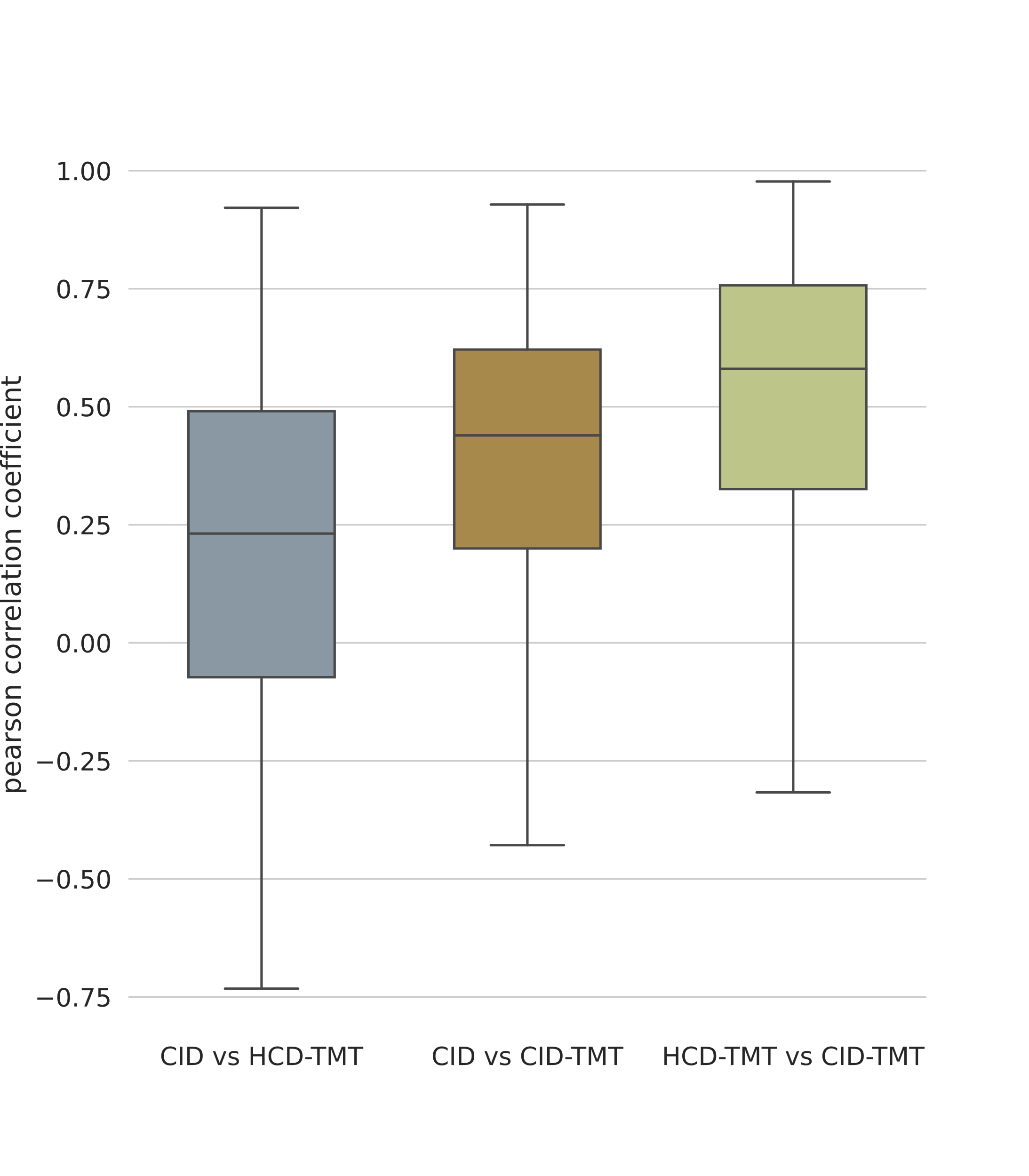


Figure S3. Distribution of Pearson correlation coefficients directly comparing observed peak intensities for the same peptidoform (not including labeling modifications) acquired with different instrument (settings) and with or without labeling: ion trap-based CID without labeling (CID), orbitrap-based HCD with TMT-labeling (HCD-TMT), and ion trap-based CID with TMT-labeling (CID-TMT).

## Supplementary data

Table S1. Training and evaluation data sets for the new MS²PIP models. The train/test data for the CID-TMT model was acquired in house.

| PRIDE Archive project | Unique peptides | Peptide type | Publication | Used for model |
| --- | --- | --- | --- | --- |
| **Train / test** | | | | |
| PXD012308 | 61 590 | Immunopeptide (HLA-II) | (Racle et al, 2019) | HCD 2021, immunopeptide HCD |
| PXD006939 | 120 427 | Immunopeptide (HLA-I/II) | (Chong et al, 2018) | HCD 2021, immunopeptide HCD |
| PXD009925 | 17 339 | Immunopeptide (HLA-I) | (Gfeller et al, 2018) | HCD 2021, immunopeptide HCD |
| PXD000394 | 134 891 | Immunopeptide (HLA-I) | (Bassani-Sternberg et al, 2015) | HCD 2021, immunopeptide HCD |
| PXD004894 | 129 011 | Immunopeptide (HLA-I/II) | (Bassani-Sternberg et al, 2015) | HCD 2021, immunopeptide HCD |
| PXD010154 (70%) | 60 598 | Chymotrypsin-digested | (Wang et al, 2019) | HCD 2021 |
| PXD041002 | 72138 | TMT-labeled, CID fragmentation, ion trap acquisition | / | CID-TMT |
| **Evaluation** | | | | |
| PXD005231 | 12 534 | Immunopeptide (HLA-I) | (Wang et al, 2019) |  |
| PXD020011 | 12 745 | Immunopeptide (HLA-II) | (Marino et al, 2020) |  |
| PXD010154 (30%) | 25 570 | Chymotrypsin-digested | (Wang et al, 2019) |  |
| PXD008034 | 35 212 | General proteomics data | (Gravina et al, 2018) |  |
| PXD005890 | 69768 | TMT-labeled, CID fragmentation, ion trap acquisition | (Hughes et al, 2017) |  |

Table S2. - The optimal hyperparameters for each new b- and y-ion MS²PIP model, as determined during hyperparameter optimization.

| Model | Eta | Max depth | Grow policy | Max leaves | Min child weight | Gamma | Lambda | Alpha | Colsample by tree | Sub-sample |
| --- | --- | --- | --- | --- | --- | --- | --- | --- | --- | --- |
| HCD 2021 (b-ions) | 0.08060612330262913 | 18 | Lossguide | 117 | 500 | 0.031142279181653326 | 0.2724553826622634 | 3.4 | 0.891381182690278 | 0.7 |
| HCD 2021 (y-ions) | 0.047107785048838 | 18 | Lossguide | 490 | 4 | 0.37528441949267444 | 0.35150807248415 | 3.3 | 0.6122042447952851 | 0.6 |
| Immunopeptide HCD (b-ions) | 0.09263630381479264 | 17 | Lossguide | 131 | 16 | 0.6048882172751935 | 0.9332236183206803 | 4.6 | 0.9898165069470042 | 0.7 |
| Immunopeptide HCD (y-ions) | 0.0594145790364741 | 17 | Lossguide | 302 | 3 | 0.03338151150211477 | 0.4430375595950531 | 4.5 | 0.9389820388602939 | 0.7 |
| CID-TMT (b-ions) | 0.09788304115318931 | 16 | Lossguide | 100 | 175 | 0.36436201158266845 | 0 | 3.1 | 0.9307205074180112 | 0.8 |
| CID-TMT (y-ions) | 0.07323226418651792 | 15 | Lossguide | 15 | 84 | 0.06487830003469364 | 0 | 0.7 | 0.7980941914509116 | 0.7 |

## Supplementary methods

### Acquisition of the in-house CID-TMT data set

Protein extracts from human ovarian cancer cell lines were digested and labelled with TMT-11 in triplicates. TMT mixes were fractionated using basic pH reversed-phase fractionation, 12 fractions were collected and analyzed in using an Orbitrap Eclipse mass spectrometer (Thermo Fisher Scientific, San Jose, CA, USA) coupled to an EASY-nLC 1000 (Thermo Fisher Scientific (Proxeon), Odense, Denmark) with a 90min gradient. Data acquisition was done using a Real Time Search MS3 method (RTS-MS3). The scan sequence began with an MS1 spectrum. In each cycle of data-dependent acquisition analysis, following each survey scan, the most intense ions were selected for fragmentation. Fragment ion spectra were produced via collision-induced dissociation (CID) at normalized collision energy of 35% and they were acquired in the ion trap mass analyzer in “Turbo” mode. MS2 spectra were searched in real time with data acquisition using the sp-human database. MS2 spectra with an Xcorr greater than or equal to 1 and less than 10 ppm precursor mas error, triggered the submission of an MS3 spectrum to the instrument. MS3 spectrum were collected using the multinotch MS3-based TMT method, in a way were ten MS2 fragment ions were captured in the MS3 precursor population using isolation waveforms with multiple frequency notches. MS3 precursors were fragmented by high energy collision-induced dissociation (HCD) at normalized collision energy of 65% and acquired in the Orbitrap analyzer.

Acquired spectra were analyzed using the Proteome Discoverer software suite (v2.4, Thermo Fisher Scientific) and the Mascot search engine (v2.6, Matrix Science). Data was searched against a customized database including the Swiss-Prot Human database plus a list of common contaminants and all the corresponding decoy entries. For peptide identification, a precursor ion mass tolerance of 7 ppm was used for the MS1 level, trypsin was used as enzyme, and up to three missed cleavages were allowed. The fragment ion mass tolerance was set to 0.5 Da for MS2 spectra. Oxidation of methionine and N-terminal protein acetylation were used as variable modifications whereas carbamidomethylation on cysteines, TMT6plex on Lysine, and TMT6plex on peptide N-termini were set as fixed modifications. False discovery rate (FDR) in peptide identification was set to a maximum of 5%. Peptides were quantified using the reporter ions intensities in MS3. Reporter ion intensities were adjusted to correct for the isotopic impurities of the different TMT reagents according to manufacturer specifications.

### Training procedure of new MS²PIP prediction models

In total, five immunopeptidomics and one chymotrypsin data set were downloaded from PRIDE (16, 17) and the CID-TMT data was generated in-house. The peptide identifications from the original data submissions were filtered at 1% FDR and converted to MS²PIP input file format. These files were passed to MS²PIP for feature vector extraction alongside the corresponding observed spectra in Mascot Generic Format.

MS²PIP accepts any peptide modifications in its input, so no prior filtering on modifications is required. Nevertheless, it is important to note that the intensity model is not aware of the presence or absence of peptide modifications. The modification information is only used to calculate the correct m/z values of (shifted) fragment ion peaks.

All models were trained with the XGBoost machine learning algorithm (20) and hyperparameter optimization was performed with the Hyperopt (21) Python package using a four-fold cross-validation evaluation scheme. The maximal number of boosting rounds was fixed at 400 and early stopping was set to 10 boosting rounds. The selected hyperparameters are listed on supplemental Table S2.

### Evaluation procedure of new MS²PIP prediction models

To evaluate the newly trained MS²PIP models, five distinct data sets were used. Firstly, the tryptic data set that was previously used to validate the 2019 HCD model, secondly a data set containing chymotrypsin digested peptides, thirdly and fourthly a data set containing HLA class I and HLA class II immunopeptides and lastly a tryptic digest acquired with CID-TMT. All data sets were parsed the same way as the training data sets. For the tryptic, chymotrypsin, HLA-I and HLA-II data sets the 2019 HCD predictions were compared to the 2021 HCD and the immunopeptide HCD model, and for CID-TMT the new CID-TMT model was compared to both current CID and TMT models. For all comparisons, Pearson correlation coefficients were computed for all singly charged peptide b- and y-ions together, with intensities square root-normalized and log2-transformed. Intensities for absent peaks in observed spectra are set to zero and are included in the comparison.
